# Best Results in Filling with Gutta-Percha

**Published:** 1900-02-15

**Authors:** B. F. Arrington


### BEST RESULTS IN FILLING WITH GUTTA-PERCHA.

To obtain the best results in filling with gutta-percha, be careful not to stuff cavities with large pieces; small pieces not more than a sixth or eighth the size of the cavity work to better advantage. Pack carefully and with force from the base of the cavity, with heated pluggers instead of heating the material. Never apply chloroform to surface of fillings. A good article of gutta-percha rightly manipulated is quite durable, and the results generally satisfactory.—*B. F. Arrington, in Dental Brief.*

